# Risk adjustment for regional healthcare funding allocations with ensemble methods: an empirical study and interpretation

**DOI:** 10.1007/s10198-023-01656-w

**Published:** 2024-01-03

**Authors:** Tuukka Holster, Shaoxiong Ji, Pekka Marttinen

**Affiliations:** 1 Finnish Institute for Health and Welfare (THL), Helsinki, Finland; 2 Aalto University, Espoo, Finland

**Keywords:** Risk adjustment, Predictive modeling, Healthcare costs, Socioeconomic information, Machine learning, Interpretation, H72, H75, I13, I18

## Abstract

We experiment with recent ensemble machine learning methods in estimating healthcare costs, utilizing Finnish data containing rich individual-level information on healthcare costs, socioeconomic status and diagnostic data from multiple registries. Our data are a random 10% sample (553,675 observations) from the Finnish population in 2017. Using annual healthcare cost in 2017 as a response variable, we compare the performance of Random forest, Gradient Boosting Machine (GBM) and eXtreme Gradient Boosting (XGBoost) to linear regression. As machine learning methods are often seen as unsuitable in risk adjustment applications because of their relative opaqueness, we also introduce visualizations from the machine learning literature to help interpret the contribution of individual variables to the prediction. Our results show that ensemble machine learning methods can improve predictive performance, with all of them significantly outperforming linear regression, and that a certain level of interpretation can be provided for them. We also find individual-level socioeconomic variables to improve prediction accuracy and that their effect is larger for machine learning methods. However, we find that the predictions used for funding allocations are sensitive to model selection, highlighting the need for comprehensive robustness testing when estimating risk adjustment models used in applications.

## Introduction

Models of healthcare costs are routinely used worldwide in applications such as healthcare provider benchmarking and risk adjustment of reimbursements made to healthcare providers, health insurance plans, and local public authorities. Health insurance market regulators use risk adjustment models to adjust the payments an insurer receives for its customers, to account for the costs the insurer is expected to incur for enrolling that specific patient-mix. This is done both to reduce the financial risk that insurers face from unprofitable customers and to mitigate selection incentives against high-expected-cost customers when the insurers are restricted in premium setting by the regulator [1–3]. In public healthcare systems, such as those of England and Finland, risk-adjusted formula funding is used by the state (or the central planner of the NHS) for allocating budgets to local authorities responsible for organizing service provision [4–6]. Formula funding is used because it allows the government to have control over total expenditure, and adjusting for risk, or *need*, is meant to ensure horizontal equity in funding so that citizens of all local health authorities have similar access to services of similar quality [5–7]. The government could alternatively use a simple fee-for-service reimbursement or allow the local authorities to collect taxes themselves but may be unwilling to devolve control of healthcare spending. A mix of different funding methods could also be used.

In Finland, healthcare is mostly organized and produced by the public sector. Geographical regions (before 2023, smaller units called municipalities) are the local health authorities that have the responsibility for providing health services for their residents, and for the most part, they also produce these services themselves. Healthcare is also provided in the occupational healthcare, funded by employers and the employed. There is a National Health Insurance system that reimburses expenses of outpatient drugs (not provided by the regional healthcare) and a small proportion of the expenses of private healthcare. Occupational and private healthcare provide mostly ambulatory primary care and specialist consultation. Private health insurances have also seen growth in the recent years. These alternative sectors cover to some extent the weak points of the regional public healthcare system, but it still has by far the largest role [8].

Funding of regions is based mainly on statistical risk adjustment models [9, 10] and at least in the beginning, regions are not allowed to collect taxes themselves. They can collect user fees, but maximum fees are limited by legislation to a low level and because of this, out-of-pocket payments have only a small role in the overall funding. In 2023, the amount of funding allocated through the need-adjusted funding formula is around 16 billion euros and is expected to grow annually. Due to the large amount allocated, even slight differences in risk adjustment models lead to significant differences in funding. Because of the size of the allocative problem and its direct impact on equity and health of the population, the development of the funding formula warrants further attention.

There is an extensive literature on risk adjustment methodology. In practical applications, ordinary linear regression is usually used to model expected service utilization statistically as a function of risk adjuster variables [11]. Effective risk adjustment has proven to be elusive because of the complexity of the factors affecting individual healthcare utilization, and empirical studies find selection incentives and resulting strategic behavior to remain in health insurance markets despite of risk adjustment [1, 12–16]. While selection incentives are not an issue for risk adjustment models used for allocating funding to local authorities, the failure of a model to compensate a local authority sufficiently is an indication of failure of horizontal equity. Insufficiently funded local authorities would be forced to lower the quality or availability or services, for example by closing local health centers.

If the policymaker is unwilling to completely abandon formula funding, there are two obvious remedies: increasing the number of risk adjuster variables in the model or improving the function linking the outcome to risk adjuster variables. The models usually use many variables on individual characteristics, such as morbidity, age and gender to predict future utilization of services, measured by costs. The variables are chosen to minimize the out-of-sample prediction error while still satisfying other heuristic constraints, such as requiring that the explanatory variables are relatively fixed characters of the individual and not subject to choices of the organization receiving the risk-adjusted payments [17]. This is to preserve the power of cost containment incentives and may rule out using variables such as prior utilization and ambiguous diagnoses as predictors.

As these constraints and data availability limit the choice of the variables, improving the functional form remains a significant potential avenue for improvement. Recently there have been some studies applying advanced machine learning methods to the problem [18–23]. We contribute to this literature by comparing linear regression to machine learning methods with Finnish data. This allows for assessing the potential of machine learning methods in the context of risk adjustment and benchmarking the OLS linear regression used in practice. Our results add further evidence to the international literature on risk adjustment.

Our other significant contribution is evaluating the effect of including individual-level socioeconomic variables in risk adjustment models. Our data contains individual-level socioeconomic information not readily available in other countries, including household income, employment status and educational attainment. While area-level socioeconomic information is available in many countries and is routinely used in studies both in the linear regression framework and with machine learning methods [4, 20], there have been few studies using individual-level socioeconomic variables [24, 25]. In addition to being less precise information, area-level variables suffer from being subject ecological fallacy [6, 20]. This means that they may pick up the effect of supply-side variables instead of need variables, leading to unwanted compensation for higher availability of services. To our knowledge, only [51] uses machine learning methods with individual-level socioeconomic variables. However, their focus is on using regression trees for identifying interactions, not on comparing predictive performance of linear regression and sophisticated machine learning methods.

Machine learning methods suffer from being “black-boxes” and, therefore, less interpretable by policymakers and less transparent to insurers, local authorities and the public. While the requirement of easy interpretability may continue to hinder the use of more complicated models in funding formulas, knowing how much our overall preferred model falls short of the optimal predictive model is important for balancing the trade-off between interpretability and predictive performance: in the literature, it is recommended to search for the “performance ceiling” for the dataset using flexible models before possibly settling on the simplest model that performs relatively well [26, 27]. The requirement of interpretability also differs by application, and black-box solutions may be more acceptable when, for example, adjusting benchmarking outcomes for patient characteristics. However, we showcase graphical methods that enhance the interpretability of machine learning methods.

## Data and empirical methods

### Data and study population

We have data on all permanent residents of Finland in 2017, which yields a population of around 5.5 million observations. We take a 10% sample of this population for computational reasons, consisting of 553,675 observations. We split this sample to separate estimation and validation samples. We split the dataset into estimation and validation samples with the ratio of 80:20, estimate the models in the estimation sample, and test the performance in the validation sample. Many studies estimating models of healthcare costs utilize quasi-Monte-Carlo designs for evaluation [23, 28, 29]. However, we use a simple split sample design to save computational time, which Kuhn and Johnson [26, 27] note to be reasonable with large sample sizes, such as ours.

**Fig. 1:**
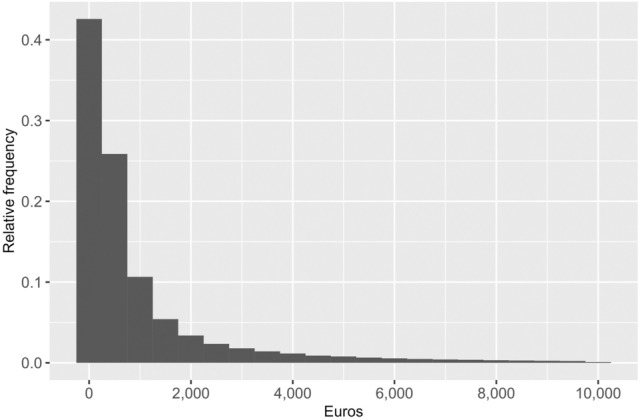
Distribution of healthcare costs in 2017, whole sample (*N* = 553,675)

We collect data on demographics and socioeconomic status on individual level from Statistics Finland, data on public healthcare utilization from the national Care Registries for Healthcare maintained by the Finnish Institute for Health and Welfare (THL), and diagnostic data from a large variety of sources. Our measure of healthcare utilization includes inpatient and outpatient visits from specialized and ambulatory regional public healthcare. Both somatic and mental healthcare are included, but social services such as assisted living and long-term care of the elderly and the disabled are not included. Visits are transformed into costs with national unit costs and summed up [10, 30]. Figure 1 plots the distribution of costs (censored at 10,000 euros) in the whole sample and it shows the high skewness that is usual in healthcare costs: while most have no costs at all, a small proportion have very high annual costs.

In our selection of explanatory variables, we seek to mostly follow the choices made in the funding models [9, 10]. All the variables used in the models are reported, and we did not have a preliminary analysis stage that would have led us to discard any unreported variables. Our measures of socioeconomic status include household income, mean income in the postal code area, the proportion of population unemployed in the postal code area, and binary indicators for whether the person was employed, unemployed, on a disability pension, a student, a conscript, with intermediary education or highly educated. Education here refers to the highest level so far obtained and corresponds to levels 3–4 of ISCED 2011 for intermediary education and levels 5 and higher for higher education [31]. We measure income as household disposable income divided by the adjusted OECD income equivalence scale. Table 1 presents descriptive statistics of healthcare costs and predictor variables, excluding the disease variables. In the table, we show the statistics separately by sample and include various percentiles of the healthcare cost distribution. The percentiles of the distribution show that the estimation and validation samples are reasonably similar. Table A1 in the appendix includes additional details on the included variables.

**Table 1:** Descriptive statistics

	Whole sample	Estimation sample	Validation sample
Variable	*N* = 553,675	*N* = 442,940	*N* = 110,735
Healthcare cost			
Mean (SD)	1,699 (6,059)	1,700 (6,109)	1,693 (5,853)
10th percentile	0	0	0
20th percentile	40	40	40
30th percentile	120	120	123
40th percentile	236	235	238
50th percentile	374	374	376
60th percentile	572	572	575
70th percentile	890	890	890
80th percentile	1538	1538	1537
90th percentile	3547	3548	3536
99th percentile	23,740	23,680	23,980
Age, mean (SD)	43 (24)	43 (24)	43 (24)
Income, mean (SD)	27,428 (32,228)	27,434 (33,044)	27,405 (28,734)
Proportion unemployed, mean (SD)	11.5 (4.4)	11.5 (4.4)	11.5 (4.4)
Mean income, mean (SD)	24,017 (4,417)	24,018 (4,424)	24,015 (4,390)
Female, *n* (%)	280,323 (51%)	224,158 (51%)	56,165 (51%)
Employed, *n* (%)	231,417 (42%)	185,115 (42%)	46,302 (42%)
Unemployed, *n* (%)	30,261 (5.5%)	24,333 (5.5%)	5928 (5.4%)
Student, *n* (%)	39,788 (7.2%)	31,774 (7.2%)	8014 (7.2%)
Conscript, *n* (%)	745 (0.1%)	592 (0.1%)	153 (0.1%)
Disability pension, *n* (%)	21,236 (3.8%)	17,059 (3.9%)	4177 (3.8%)
Intermediate education, *n* (%)	187,244 (34%)	149,875 (34%)	37,369 (34%)
Higher education, *n* (%)	142,759 (26%)	114,062 (26%)	28,697 (26%)

The table presents mean (SD) for continuous variables and n (%) for categorical variables, separately for the whole sample, estimation sample and validation sample. For the predicted variable healthcare cost, also percentiles of the distribution are presented. Of predictors, only demographic and socioeconomical variables are presented. Summary information of disease variables are in the appendix

The disease classification system we use has been developed specifically for need-adjusted regional funding in Finland. It contains 51 categories and is based on clinical expert knowledge and data analysis. An important special feature of the Finnish classification system is that it uses diagnostic information from multiple sources and because of that, may be more robust compared to classifications using only inpatient diagnoses, for example. Our diagnostic data come from a comprehensive collection of national registries: in addition to inpatient diagnoses, we utilize diagnoses from outpatient visits, a few disease-specific registries (the Finnish Cancer Registry, the Visual Impairment Registry, and the Medical Birth Register), registries concerning disability pensions and sickness allowances provided by the Social Insurance Institution (SII) and the Finnish Centre for Pensions (ETK). Table A2 in the appendix contains descriptive statistics for the disease variables. Table A3 summarizes our disease variable definitions, most importantly the mappings to ICD-10.

### Statistical analysis

We estimate risk adjustment models, where the outcome is total annual public healthcare expenditure from 2017 and the explanatory variables are from 2016 to 2017. The model is a hybrid of prospective and concurrent risk adjustment models, as recommended by Dudley et al. [32]. This means that while most explanatory variables are from year 2016, some (socioeconomic background variables, childbirth) are from the same year as the outcome variable (2017). Our models include linear regression and three ensemble learning methods discussed below. Ensemble learning algorithms, or ensemble methods, combine results from multiple machine learning models to improve predictive performance. We estimate ensemble learning models that use regression trees as the underlying model [33]. We implement analyses in an R environment with packages *ranger*, *gbm* and *xgboost* [34–36]. We apply three standard evaluation metrics in the validation sample to evaluate the model performance: Root-Mean-Square-Error (RMSE), Mean Absolute Error (MAE), and R-squared (*R*^2^).

Machine learning models have *tuning parameters* (or hyperparameters) that allow the model to fit the data flexibly. A central challenge in choosing the best predictive model is that of *overfitting*: the model that fits the estimation sample the best is unlikely to have good performance in future samples, the prediction of which is the objective of the model [26]. To avoid overfitting, optimizing the tuning parameters requires estimating the out-of-sample error. We use the standard procedure of fivefold *cross-validation* to simulate the out-of-sample error and to choose the optimal hyperparameter values. In *n*-fold cross-validation, the estimation sample is randomly divided into *n* subsamples of equal size. Of the *n* subsamples, *n-1* are used as a single estimation sample to estimate the parameters of the model. One is left as a validation sample, in which the model is applied to calculate the difference between the predicted and the observed values. This is done *n* times until each of the *n* subsamples has been used as the validation sample once. Finally, an average of the estimated errors is calculated. The number of combinations tested is, in practice, limited by computational resources. For random forest and GBM, we used grid search with around 80 combinations. For XGBoost, 240 combinations were tested.

Regression trees sequentially partition the sample into mutually exclusive subsamples by splitting the sample based on values of the predictor variables. The predicted value of the outcome is the average outcome in the subsample. The resulting model has a tree structure: in the first stage, the whole sample is split to two subsamples, and in the following stages the subsamples are similarly split. Compared to linear regression, regression trees have the advantage of not requiring explicit modeling of the functional relationship between the outcome and the predictor variables and they naturally accommodate for non-linearities of the data. However, single regression trees tend to be unstable with respect to small changes in the data, and usually do not perform very well out-of-sample [26].

In random forests, several bootstrap samples are generated from the original sample and a complex tree is grown in each one [37]. A random subset of the predictor variables is selected at every split of every bootstrap sample, and the best split among the predictors in the subset is picked. This has the advantage of further reducing the variance of the prediction by reducing the correlation between the trees. The final prediction is the average prediction across individual trees. The central tuning parameter of the random forest is the number of predictors sampled *m*. The fraction of the estimation sample picked for the bootstrap samples can also be varied. The complexity of the trees is controlled by the minimum number of observations in terminal nodes and the maximum number of terminal nodes. The number of trees (the number of bootstrap samples generated) must also be decided, but it is shown that random forests are not prone to overfitting as the number of trees grows: the standard advice is to use at least 1000 trees [37].

The gradient boosting machine is an algorithm that iterates the prediction over a pre-specified number of rounds [38, 39] In every iteration, a model is fit to the residuals of the model estimated in the previous step, and the prediction is the sum of the current predicted value and the predicted value from the previous step, thereby trying to reduce the errors from the previous step. Whereas a random forest utilizes ensembles of complex unpruned trees, GBMs use simple trees to sequentially build the model. Simple (or shallow) trees are computationally cheap to fit and GBMs avoid overfitting by learning slowly. The tuning parameters of the GBM include tree depth, the minimum number of observations in a terminal node, the fraction of estimation data selected at every iteration, the learning rate and the number of iterations (or trees). The learning rate determines the fraction of the current iteration’s predicted value added to the previous predicted value.

XGBoost is an extension of the gradient boosting machine that improves the computational efficiency of the algorithm while considerably enhancing model flexibility [40]. Unlike standard GBM, XGBoost includes tree pruning, regularized objective function and additional options for randomization. Splits are made up to the specified maximum tree depth, after which the pruning of the tree starts. In regularized boosting, the objective function optimized at the tree construction stage includes a penalty term for complexity (i.e., the number of splits). As random forest, XGBoost allows for sampling a fraction of the predictor variables for tree construction. In health economics literature, XGBoost has been applied before in the context of mortality prediction [41]. In addition to those of standard GBM, XGBoost offers a rich set of tuning parameters for model customization. The most important tuning parameters are the two regularization parameters that penalize for complexity and the fraction of predictor variables used in constructing the tree of the current iteration.

### Model interpretation

We adopt three techniques for model interpretation, including *predictor variable importance* to understand which variables contribute most to the model prediction, *partial dependence plots* (PDPs) to investigate how the model responds to changes in specific variables, and *local interpretations* about how the predicted value is composed for a single observation. All the model interpretation graphs are drawn on the estimation sample.

Variable importance score helps to measure the relative influence of specific variables on the outcome. We opt for the standard method of relative influence, where at each split of each tree, the decrease in the mean squared error is calculated. For every variable, the average of the decrease achieved over all splits (over all trees) is calculated. The variable importance scores are then sorted according to the value of average decrease, i.e., the variable with the largest average decrease is regarded as the most important one. The resulting variable importance measure gives an indication of the relative magnitude of the association between the outcome and the predictors. However, variable importance does not reveal whether the association between the predictor and the outcome is negative or positive and cannot be interpreted in the same way as regression coefficients. We use the implementation from the R package *vip* [42].

The second interpretation technique is Partial Dependence Plots that plot the average predicted value as a predictor variable of interest varies over its marginal distribution [38]. For each observation in the estimation sample, the predicted value is calculated for every unique value of the predictor variable, while other predictor variables are kept constant. For each value of the predictor variable, the average prediction over all observations is calculated and is then plotted against values of the predictor variable. Partial dependence plots then show what the expected healthcare cost at the predictor variable value specified on the x-axis would be, if every individual in the sample were to have that predictor value. This allows for assessing how the predictor variable contributes to the prediction at different values of its distribution. We utilize the implementation from the R package *pdp* [43].

These methods of overall model interpretation can be complemented with methods of local interpretation, in which the model fit is assessed locally around the individual. Local Interpretable Model-agnostic Explanations (LIME) is a visualization method for local interpretation that fits a linear approximation to the model in the neighborhood of the specified observation [44]. The coefficients of the local linear model allow for interpreting the behavior of the more complex model in that locality, including the signs of the associations between the outcome and the most important predictors.

Obviously, with samples running to millions of observations, as is common in risk adjustment applications, the importance of individual observations is small. However, LIME allows the policymaker to observe how the model behaves for a hypothetical observation with some pre-specified characteristics. A regulator of health insurance markets could find it important to understand how the model behaves for those that are highly under or overcompensated by the model [45]. This would be possible by looking at representative observations at the tails of the residual distribution of the model. Suppose those with kidney diseases are more likely to be in both the overcompensated and undercompensated groups. Is their probability of falling to the undercompensated and not the overcompensated group related to, for example, age, socioeconomic situation or presence of comorbidities? For transparency, health insurers could also be given access to local interpretations. Policymaker designing formula funding of public services may have similar concerns: for some interesting representative individual, how would changes in predictor variables of interest affect the prediction and finally, the funding allocated based on that individual? We use the implementation from the R package *lime* [46].

## Results

### Prediction Results

Panel A of Table 2 presents the main results with the linear regression, random forest, GBM, and XGBoost with all variables. The results show that ensemble methods are better than the linear model with lower prediction errors and higher *R*^2^ scores, with *R*^*2*^ values of around 0.162–0.17 for ensemble methods and 0.1368 for linear regression. XGBoost with an improved boosting algorithm slightly outperforms the classic GBM, and the random forest algorithm achieves the best RMSE and R-squared scores. The different measures are not in complete agreement: in terms of MAE, which does not give as high a weight to large values as RMSE and *R*^*2*^, GBM and XGBoost outperform random forest (around 1,760 to 1,779) by much more than random forest outperforms linear regression (1779 to 1782). Much of the higher performance by ensemble learning seems to be due to it being better in predicting the outliers, as truncating or censoring healthcare cost at 99th percentile (24,000 euros) decreases the gap between ensemble learning and linear regression (results not shown).

**Table 2:** Model comparisons summary

Method	RMSE (↓)	MAE (↓)	R-squared (↑)
Panel A: results with all variables			
Linear regression	5592.04	1782.46	0.1368
Random forest	5482.86	1779.21	0.1702
GBM	5509.75	1762.36	0.162
XGBoost	5504.32	1760.31	0.1637
Panel B: results without socioeconomic predictor variables			
Linear regression	5603.47	1790.01	0.1333
Random forest	5614.97	1784.05	0.1297
GBM	5558.53	1774.37	0.1471
XGBoost	5553.43	1768.13	0.1487
Panel C: results with only socioeconomic variables as predictors			
Linear regression	5927.39	2097.41	0.0302
Random forest	5852.32	2084.97	0.0546
GBM	5850.11	2086.67	0.0553
XGBoost	5839.7	2083.5	0.0586

The table presents three standard measures of validation sample error for all the four models estimated using: all the predictor variables (panel A), without the socioeconomic predictor variables (panel B), with only the socioeconomic predictor variables (panel C). An upward pointing arrow indicates that higher is better and a downwards pointing arrow indicates that lower is better

**Fig. 2:**
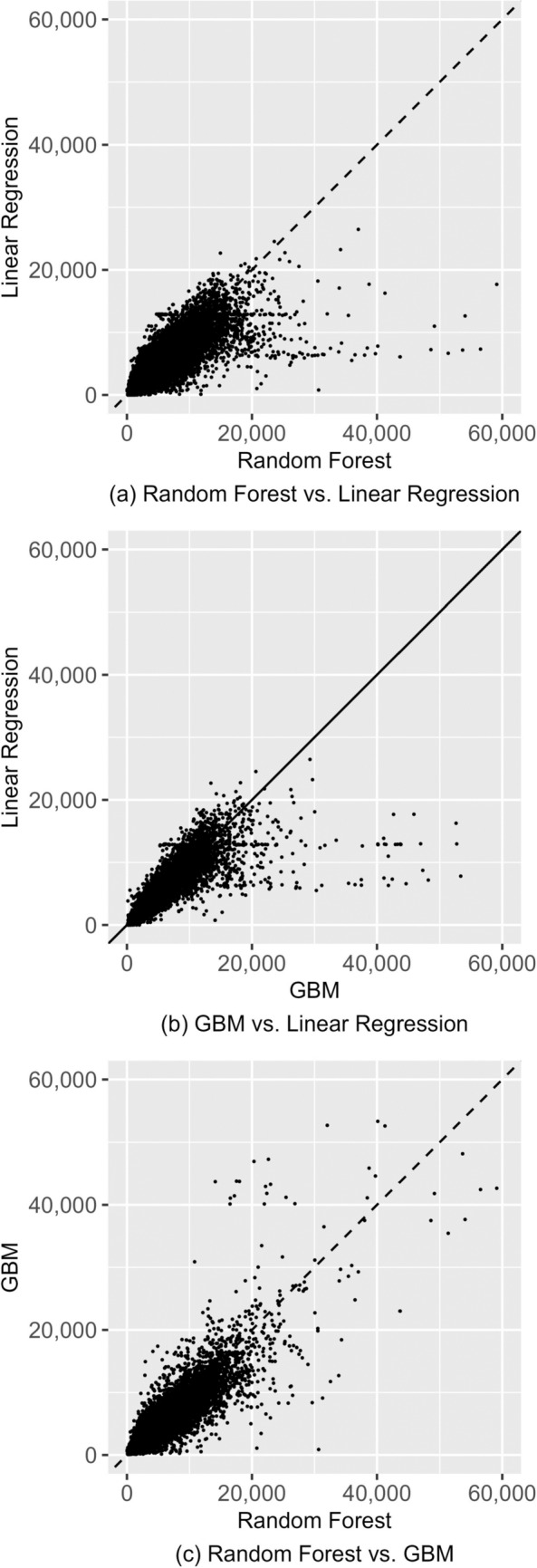
Model predicted values plotted against each other, validation sample

**Fig. 3:**
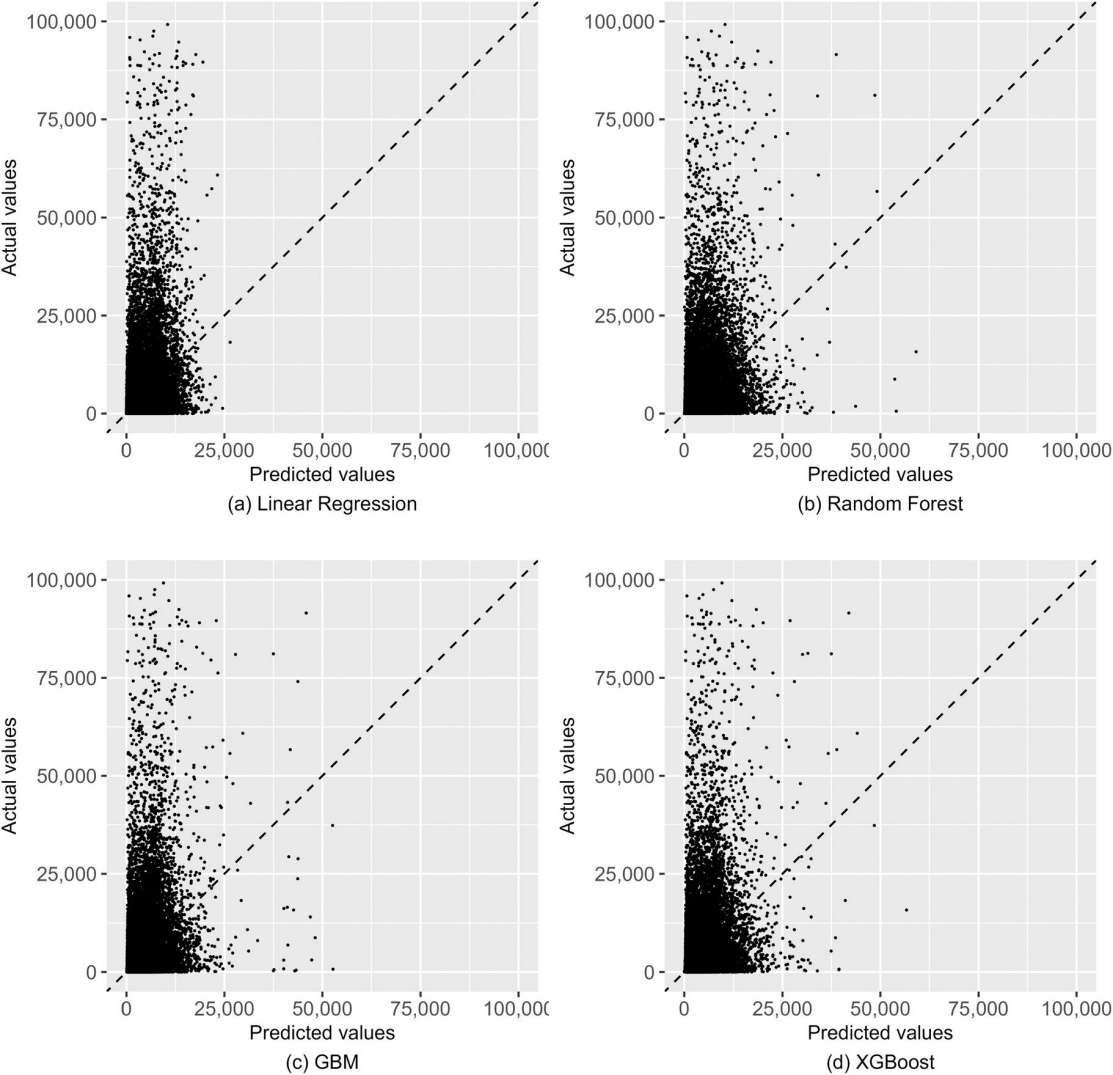
Model predictions plotted against the actual values, validation sample

Figure 2 plots the predicted values from the models against each other (XGBoost is not shown for parsimony, as results from it are very similar to those from GBM). Observations for which the predicted values from the models agree fall on the diagonal line. The figure shows that random forests and GBM tend to produce much higher predictions than linear regression, but those from random forests and GBM more closely resemble each other. Figure 3 plots the predicted values against actual observed costs and shows that none of the models seem to do well in predicting the actual values: only a very small proportion of the predictions fall on the diagonal. It also again shows that linear regression has a much lower tendency to produce high predictions. While low predictive performance is known to be usual in risk adjustment models [11], Fig. 3 provides quite a striking visualization of this.

We also present predictive performance with only morbidity, age and gender variables (Table 2, panel B) and only socioeconomic information (Table 2, panel C) to showcase the additional predictive power obtained from individual-level socioeconomic information. Comparing the results shows that socioeconomic information can significantly improve model performance but there are also some surprises. When socioeconomic variables are excluded, random forest performs the worst. Consequently, with random forests, the improvement in the proportion of out-of-sample variance explained is the largest and R-squared rises by over 30%. With linear regression, the additional explanatory power of socioeconomic information is relatively low, with R-squared rising only from 0.1333 to 0.1368. For GBM and XGBoost, the improvement is significant but less so than for random forest. As tree-based methods take interactions between predictor variables implicitly into account and linear regression does not [26], these results seem to indicate that there are significant interaction effects between socioeconomic, disease and age variables for predictions of healthcare costs. Panel C shows that when only socioeconomic predictors are used in the models, the relative performance of linear regression is even lower than when all the variables are considered. It is also evident that on their own, socioeconomic variables are not able to explain a very significant portion of the out-of-sample variance.

**Fig. 4:**
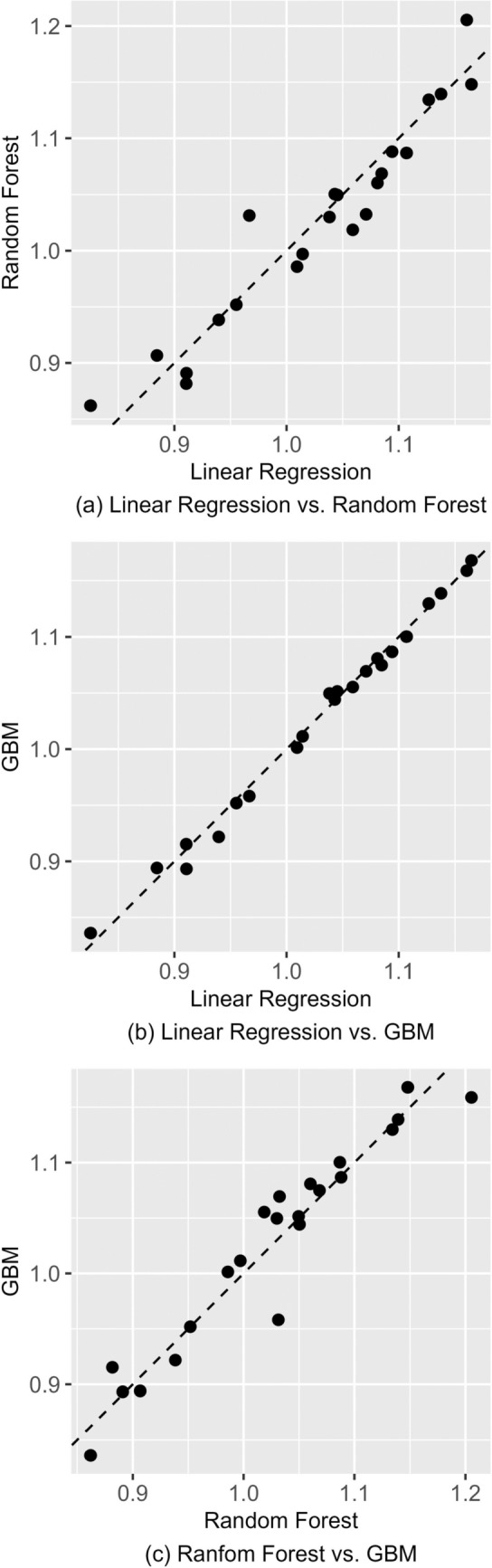
Need multipliers calculated with different models plotted against each other, validation sample

In Table 3, we compare the so-called regional *need multipliers* calculated using the predictions of the models. The multiplier is calculated by taking the mean predicted cost in the region in the validation sample and dividing it by the validation sample mean of predicted cost for the whole country. In effect, the need multiplier tells us the relative average need of the region compared to the national average. This is a convenient way of presenting the variation in predicted need across the country and is directly used in the actual funding formula. The need multipliers then allow for straightforward comparisons of results from different models. Figure 4 plots the need multipliers calculated with different models against each other (XGBoost not shown).

**Table 3:** Need multipliers for regions

Region	Linear regression	Random forest	GBM	XGBoost
Southwest Finland	1.019	1.006	1.007	1.005
Satakunta	1.053	1.042	1.063	1.059
Kanta-Häme	1.062	1.008	1.049	1.046
Tampere Region	1.000	0.988	1.002	1.002
Päijät-Häme	1.098	1.104	1.097	1.096
Kymenlaakso	1.135	1.142	1.139	1.136
South Karelia	1.091	1.078	1.107	1.110
Southern Savonia	1.166	1.147	1.17	1.165
Northern Savonia	1.129	1.129	1.129	1.129
North Karelia	1.172	1.204	1.191	1.184
Central Finland	1.019	1.006	1.009	1.02
South Ostrobothnia	1.099	1.077	1.083	1.076
Ostrobothnia	0.956	1.032	0.957	0.966
Central Ostrobothnia	1.061	1.091	1.077	1.070
North Ostrobothnia	0.997	0.985	0.988	0.989
Kainuu	1.161	1.161	1.149	1.154
Lapland	1.096	1.121	1.111	1.108
Helsinki	0.872	0.892	0.884	0.884
Vantaa and Kerava Region	0.899	0.865	0.895	0.896
Western Uusimaa	0.799	0.835	0.808	0.812
Eastern Uusimaa	0.979	0.968	0.954	0.953
Central Uusimaa	0.911	0.882	0.897	0.892

The table presents regional need multipliers calculated from all four models. Need multipliers are calculated by dividing the regional mean predicted cost by the national mean predicted cost. For the whole country, the need multiplier is therefore 1. The need multipliers presented here are calculated in the validation sample

The results show that model selection does matter. In general, GBM and XGBoost seem to produce need multipliers that are relatively close to those given by the linear model, even though they have much better predictive performance by any metric. In panel B of Fig. 4, which plots need multipliers from GBM against those from linear regression, the points fall reasonably close to the diagonal. However, panels A and C of Fig. 4 contrast random forests to the other models and show much more variation. For some regions, random forest would result in allocations significantly different from those given by other models. This is noteworthy, as random forest is the best performing method in terms of *R*^*2*^ and RMSE. There are some exceptions to the general rule: for Eastern Uusimaa, the need multiplier from the random forest is closest to that of the linear model, as the gradient boosting machines give much lower values. As would be expected, GBM and XGBoost offer mostly very similar results.

As a back-of-the-envelope calculation, the amount allocated to a region through the model can be calculated as the need multiplier times the number of people in the region times the amount per person allocated through the model. With 16 billion euros allocated and around 5.5 million citizens in Finland, the funding per person is around 2900 €. There are 660,000 citizens in Helsinki, so for it the linear model would allocate 0*.*872 ∗ 660,000 ∗ 2900 ≈ 1669 million euros. Similar calculations give 1707, 1692 and 1692 million euros for the random forest, GBM and XGBoost, respectively. Though the differences are in tens of millions of euros, these allocations are quite close to each other in relative terms. If we take the region of Ostrobothnia with 176,000 citizens, we get 488, 526, 488, 493 million euros for the linear model, random forest, GBM and XGBoost, respectively. Here, the difference between random forest and the other methods is striking.

As our validation sample consists of only 100,000 observations and contains only a few thousand observations for the smallest regions, it is possible that our results are due to sample-specific random variation that would disappear with larger samples. Because of this, as a robustness test, we calculated predictions from the models for the whole sample of around 550,000 observations. While the exact need multipliers differ by the sample, our general result holds: the predictions are not very robust to model selection.

### Interpretations

**Fig. 5:**
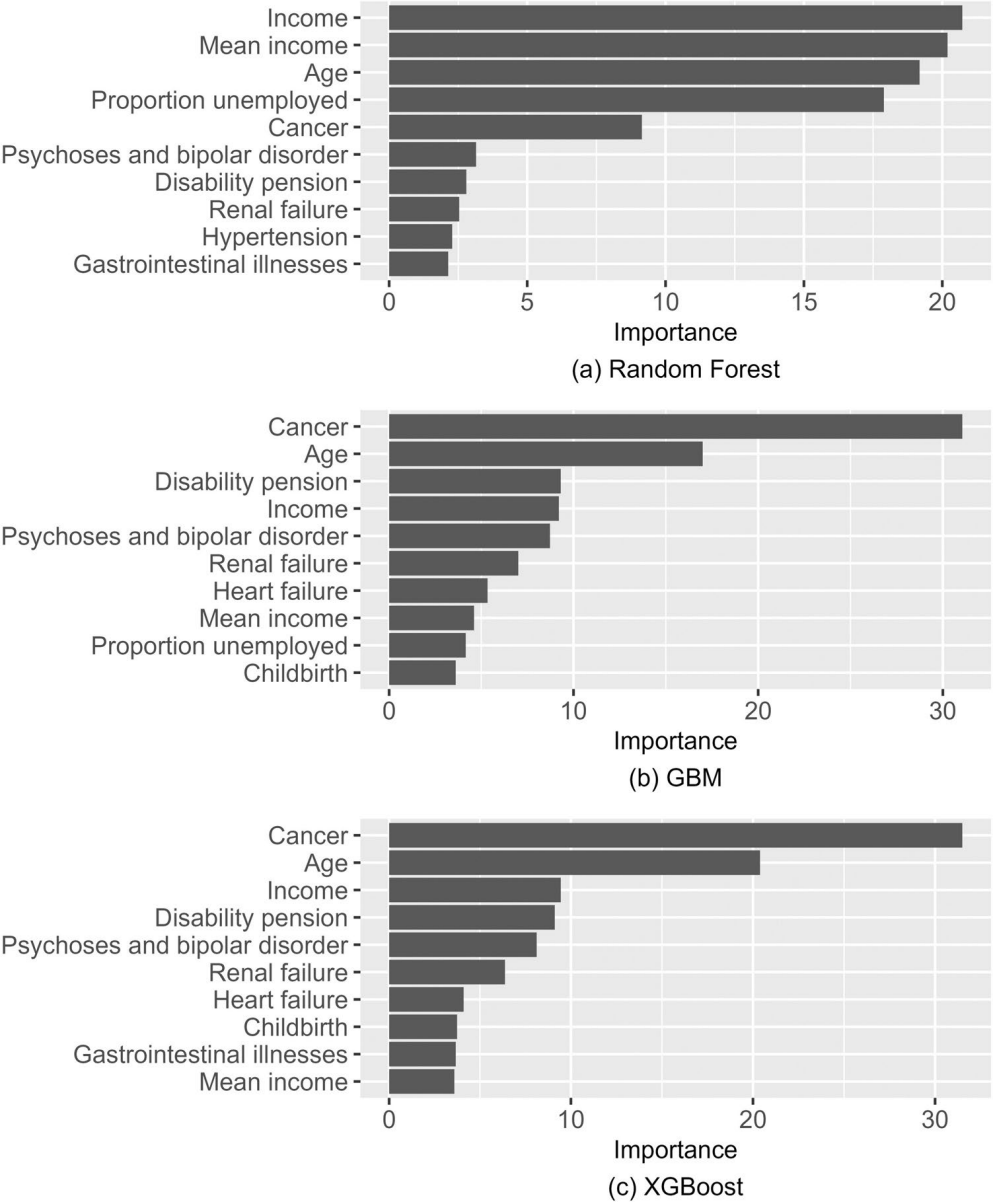
Predictor variable importance of (**a**) random forest, (**b**) GBM and (**c**) XGBoost, estimation sample

We show the variable importance values in Fig. 5, which shows the relative importance score of the top ten most important variables for the three ensemble methods. Cancer and age are the most important and the second most important variables for cost prediction in both XGBoost and GBM. They agree closely also on the relative importance of the other top ten variables: while their relative weights differ somewhat, the same variables are selected. Random forest takes household income as the most important, mean income in the postal area as the second most important and places age as the third important variable. As was seen above in Table 2, random forest benefits the most from the inclusion of socioeconomic variables, and this is clearly reflected in the variable importance scores. The unemployment rate in the postal area was ranked fourth, which was not in the top ten for the other models. Of the disease variables, cancer, psychosis, gastrointestinal diseases and renal failure are included in the top ten for all three models. Age, disability pension, income and mean income in the postal area are also in the top ten for all models. To conclude, the models are in reasonable agreement over the most important variables, though relative weights differ.

**Fig. 6:**
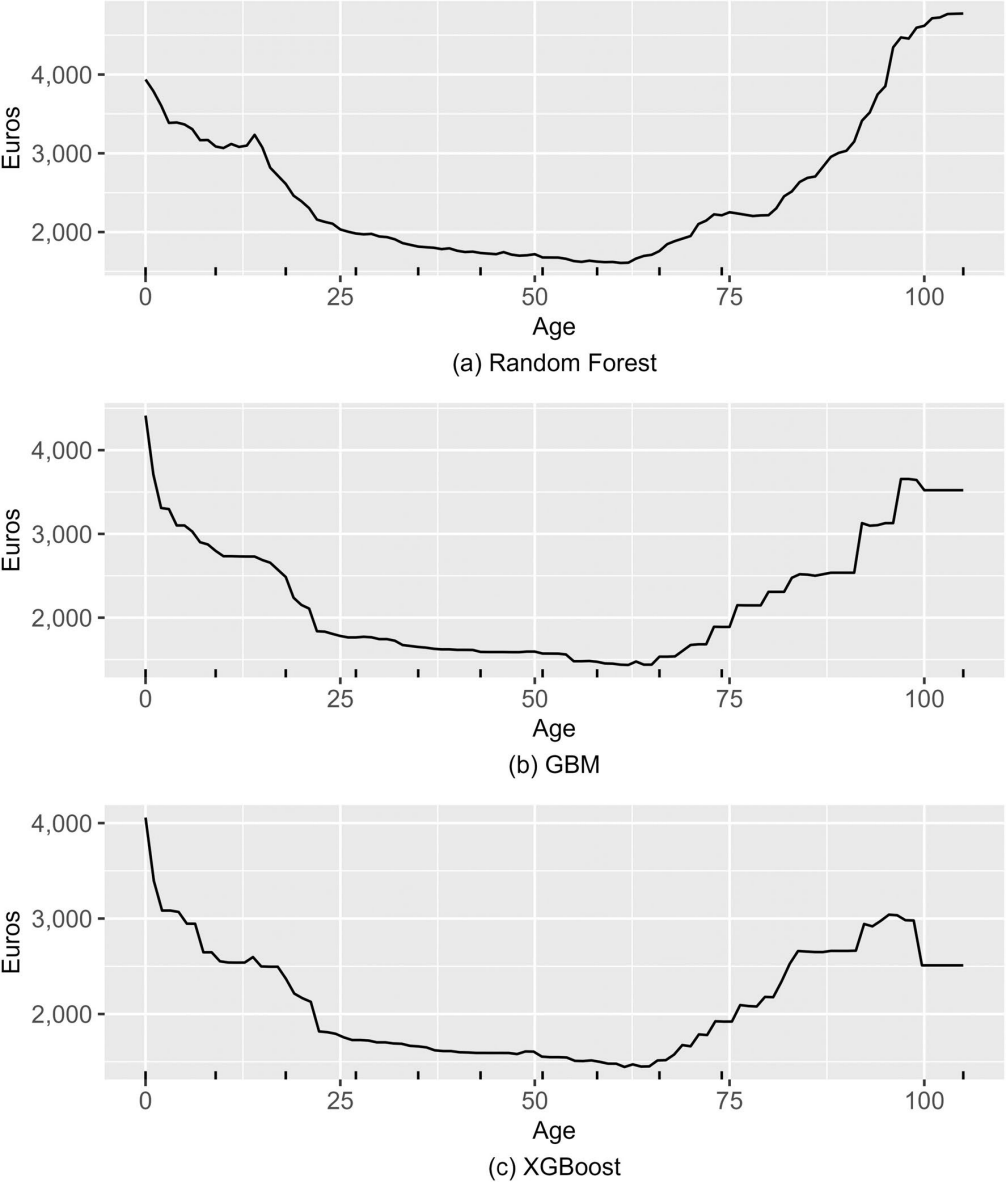
Partial dependence plots of (**a**) random forest, (**b**) GBM and (**c**) XGBoost, estimation sample

We choose the age variable, which is recognized as one of the most important variables by all the ensemble learning methods, to draw the partial dependence plots (Fig. 6). Going back to the definition of a partial dependence plot, note that here age is varied between 0 and over 100 for each observation, while other predictor variables are kept constant. The predictions from GBM and XGBoost respond to the age variable in a very similar way: the relation between age and healthcare cost is U-shaped with infants and the elderly using comparatively more healthcare when compared to the young and the middle-aged. For the very old, there is a decline in the predicted cost. For random forest, the shape of the curve is otherwise similar but the increase in predicted cost for years after age 65 is substantially steeper and remains monotonic. The PDP from random forest is therefore more in line with the conventional understanding of the relation between age and healthcare cost. It seems likely that GBM and XGBoost are less robust to the outliers here, as there are under 80 persons aged 100 or over in the estimation sample.

**Fig. 7:**
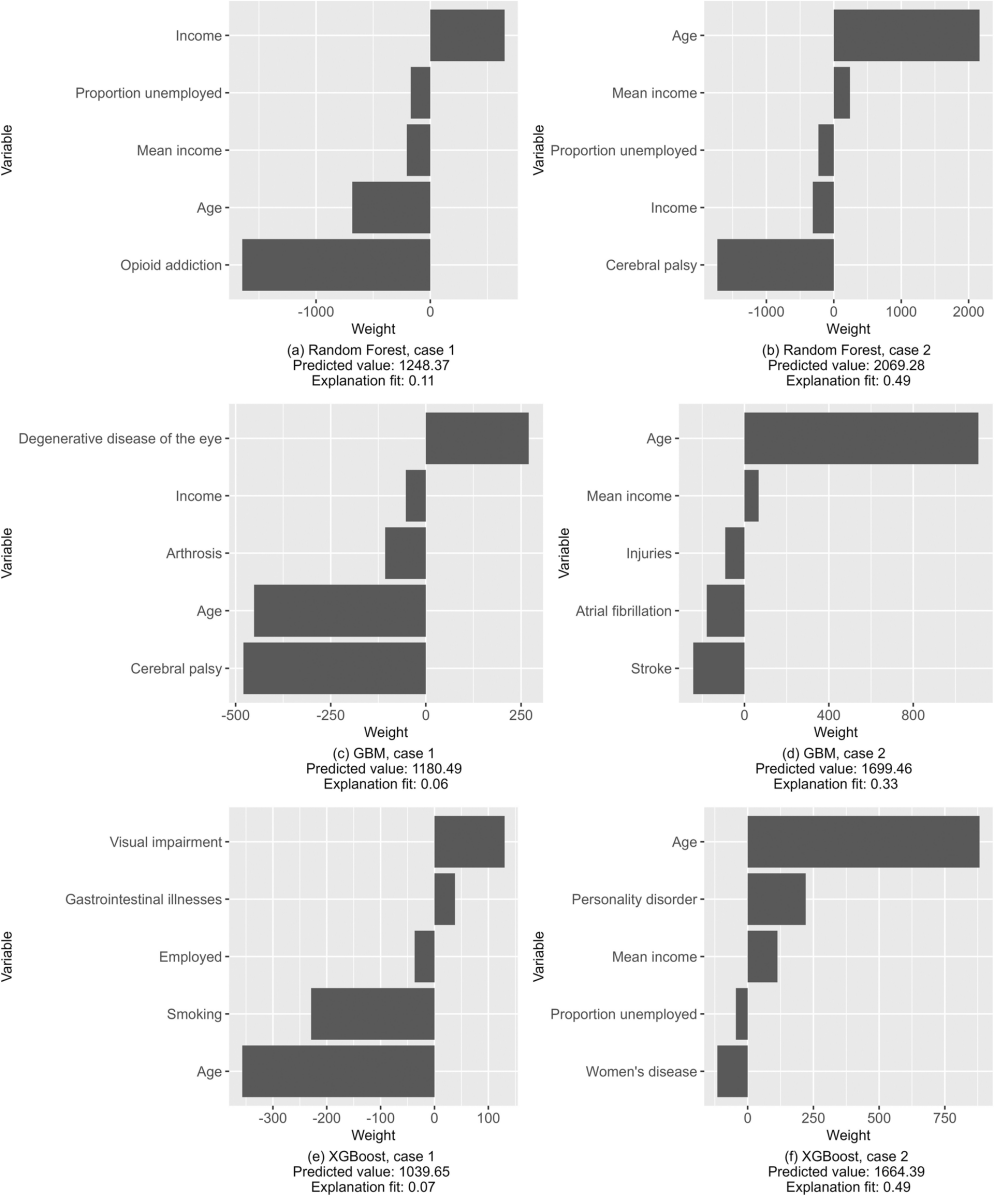
LIME explanations of (**a**) random forest, (**b**) GBM and (**c**) XGBoost, estimation sample

Last, we illustrate the local interpretations with LIME in Fig. 7, which shows both the positive and negative impact of the top-5 most important variables on the prediction. We construct two fictional observations by randomly sampling variable values from the actual observations (labeled as cases 1 and 2). Actual observations are not used to avoid disclosing sensitive information. For case 1, age has a negative effect in all three models, while it increases the predicted value for case 2 in all the models. For the other variables, there seems to be little agreement between the models, and the disease variables used by the models differ. Explanation fit is very low for case 1, indicating that the local linear approximation used by LIME does not approximate the complex ensemble model well. For both cases but especially for case 2, the predicted values vary significantly between the models. While the models are in reasonable agreement over the variables that have the largest overall effect (Fig. 5), their behavior around individual observations seem to be very different and is not easily captured by a local approximation. This can reflect fundamental differences between the models but may also be the result of our data, where a relatively sparse number of mostly binary variables is used to explain a highly skewed data with high variance.

## Discussion

Our results suggest that advanced machine learning methods significantly outperform linear regression, which is more in line with Vimont et al. [23] and Irvin et al. [20] than Rose [18] and Shrestha et al. [19]. In terms of out-of-sample R-squared, random forest was the best performing method and improves 25% over linear regression. This finding may be due to the grid search we perform to select the optimal tuning parameters, which may be needed for ensemble models to significantly outperform linear regression. It should also be noted that our results provide, in some sense, a lower boundary for this advantage because the ensemble models used would likely perform even better if computational power allowed for a more rigorous grid search. The results also show that the relative ranking of the ensemble learning methods varies by the evaluation metric, with RMSE favoring random forests and MAE the gradient boosting machines. As has also been noted by others [47, 48], the choice of the metric matters.

In terms of interpreting and communicating the contributions of individual predictors, linear regression remains clearly the best. Understanding the objectives of the regulator or the central planner remains paramount, and the issues of interpretability and transparency present problems for both public healthcare formula funding and risk adjustment of health plans. In public healthcare, a common perception that the health budgets are given from a black box may erode the legitimacy of the system. For health insurers, Ellis et al. [1118] have argued that weak interpretability may have the positive side effect of making risk selection more difficult, as a health insurer cannot discern the exact amounts allocated per health condition.

On the other hand, transparency regarding the compensation given per health condition may also have a directive role. To control their financial risk, an insurer and local public health authority would both want to know the compensation given for a condition. They could then try to match the future spending to the compensation. Compared to a health insurer that may restricted by binding contracts to reimburse the total spending by a patient (decided by a doctor), a public health authority may find it easier to match service provision to whatever level of available funding on the go. For example, in Finland, the legislation does not for the most part provide a specific list of services (or their quality and availability) that the regions would need to adhere to. Reasons of financial stability may then argue for the transparency of a model to be more important in risk adjustment of health plans. In any case, we believe that local interpretation methods such as LIME may hold great potential for increasing the transparency of ensemble methods, especially to health insurers wanting to understand the costliness of individual enrollees. The overall model interpretation methods can support in this and would also be useful in increasing the legitimacy of the models to the wider public.

We also show that predictions of healthcare costs differ substantially between the models and that these differences would translate into significant differences in funding allocations. Random forest was the best performing method in terms of *R*^*2*^ and RMSE, but it would also yield funding allocations that differ significantly from the other models. These results are worrying, in the sense that one would wish for a risk adjustment model to be reasonably robust to various aspects of model selection. It is understandable that models with better out-of-sample performance yield predictions that differ from those of worse models; otherwise, there would be little reason to develop better models. However, it would be reassuring to see models that are reasonably similar in terms of popular evaluation metrics, like random forest and the gradient boosting machines are in terms of *R*^*2*^, to agree on their predictions.

One criticism of the English formula funding system has been that it is problematic to base funding on statistical models with low predictive power [49]. Our results demonstrate some validity in that view: none of the models discussed in this paper do well in predicting healthcare costs, and when the models capture only a low portion of the total variance, there is room for model selection and specification to influence the results. This lack of robustness threatens to reduce the objective validity of the model and the resulting funding formula. The English NHS funding formula [4] is probably the most interesting international comparison for us, and our results obviously encourage for similar robustness analysis there. While our study considers the specific application of national health service funding, our results are of much broader interest. As analogous models are used in risk adjustment by regulators of health insurance markets, similar robustness tests should be undertaken there.

Our study has some limitations, notably we include a relatively small set of disease variables. An interesting future research question would be how a more comprehensive set of predictor variables would affect the relative rankings of the models in terms of performance, as in Iommi et al. [21]. Tree-based methods naturally consider interactions between predictor variables, which is likely to explain much of their better performance. A possible avenue of future study would be to investigate how much including explicitly defined interactions to linear regression would improve its performance. One way would be to search for interaction terms with tree-based methods, as investigated by Buchner et al. and van Veen et al. [50, 51]. Another important question would be whether a more comprehensive predictor set would lead to less variation in the predictions, achieving convergence between the models.

We also find that individual-level socioeconomic information, most importantly income, contributes significantly to the predictions for some models. The benefit from including socioeconomic variables differs significantly between models and is the largest for random forest and the lowest for linear regression, indicating strong interactions between socioeconomic status, age, and health. The propensity to use public health services is likely to be strongly influenced by access to private and occupational healthcare services, which varies with income and labor market status. Socioeconomic status is also likely to be correlated with unobserved health, which is another mechanism through which it can affect healthcare cost. While the importance of these considerations will differ by institutional setting, our results show that the inclusion of individual-level socioeconomic information should be considered where possible.
